# Neighbor-Enhanced Link Prediction in Bipartite Networks

**DOI:** 10.3390/e27060556

**Published:** 2025-05-25

**Authors:** Guangtao Cheng, Chaochao Liu, Chuting Wei, Yueyue Li, Xue Chen, Xiaobo Li

**Affiliations:** 1 School of Information Engineering, Tianjin University of Commerce, Tianjin 300133, China; weichuting@stumail.tjcu.edu.cn (C.W.); liyueyue@stumail.tjcu.edu.cn (Y.L.); 2 Chinese Academy of Cyberspace Studies, Beijing 100048, China; chaochaoliu@tju.edu.cn; 3 Law School, Tianjin University, Tianjin 300054, China; xuechen@tju.edu.cn; 4 School of Marine Science and Technology, Tianjin University, Tianjin 300054, China; lixiaobo@tju.edu.cn

**Keywords:** link prediction, bipartite networks, structure similarity, quadrangle graph

## Abstract

Link prediction in bipartite networks is a challenging task due to their distinct structural characteristics, where edges only exist between nodes of different types. Most existing methods are based on structural similarity, assigning similarity scores to node pairs under the assumption that a higher similarity corresponds to a higher likelihood of connection. Local structural methods, in particular, are widely favored for their simplicity, interpretability, and computational efficiency. However, real-world bipartite networks often exhibit highly heterogeneous node degree distributions, which introduce biases and undermine the effectiveness of traditional local structure-based methods. To address this issue, we propose a novel link prediction framework that explicitly adjusts for the degree heterogeneity of intermediate nodes between unconnected node pairs and incorporates their influence within local connection patterns formed around these pairs. Furthermore, our framework differentiates between the roles of same-type and cross-type nodes by leveraging quadrangle graphs between unconnected nodes. This approach allows for a more nuanced capture of unique properties of bipartite networks and effectively mitigates the inherent degree bias commonly observed in such networks, resulting in considerable improvements in prediction accuracy. Experimental results on ten diverse bipartite networks demonstrate that our framework achieves competitive and robust performance compared to nineteen state-of-the-art link prediction methods.

## 1. Introduction

Link prediction is a fundamental technique for analyzing relationships between entities within a network, and it has garnered significant attention across various fields of study [1,2]. The primary goal of link prediction is to identify missing connections or forecast future interactions. Due to its ability to unveil hidden mechanisms and the evolving patterns within real-world networks, link prediction has become invaluable in a wide range of practical applications. For instance, in criminal investigations, it assists in identifying concealed relationships between suspects or events [3,4]. In protein interaction studies, it supports the discovery of novel pathways that are essential for understanding cellular functions and drug design [5,6]. Additionally, link prediction has found widespread applications in recommendation systems, where it enhances personalization and improves user experience [7,8].

Traditional link prediction approaches can be categorized into similarity-based methods [9], probabilistic models [10], matrix factorization-based models [11], and network embedding approaches [12]. However, due to the distinct structure of bipartite networks, where links occur only between nodes from two different sets, traditional link prediction methods face inherent limitations and are not well suited for bipartite networks. To overcome these challenges, various methods specifically designed for link prediction in bipartite networks have been proposed, including similarity-based approaches, projection-based techniques, and dimensionality reduction methods.

Similarity-based methods [5,13] are relatively simple. They estimate the probability of a link between two unconnected nodes based on their similarity score, which is computed using the topological structure of the network. In particular, local similarity indices [9] often rely on the common neighbors (CNs) of two nodes, either in terms of the number of CNs or the topological structure of those CNs. These methods are generally straightforward and efficient as they only consider local neighborhood information of two unconnected nodes. Projection-based methods [14,15] transform bipartite networks into unipartite networks, enabling link prediction to be performed on the resulting projections. A key advantage of these methods is their ability to leverage advanced link prediction techniques designed for unipartite networks. However, the effectiveness of this approach heavily depends on the accuracy of the projection. Dimensionality reduction methods, such as matrix factorization (MF) [16] and network embedding techniques.

Ref. [17], aim to reduce the complexity of the data while preserving its underlying structure. Similar to projection-based methods, dimensionality reduction approaches must retain as much topological information as possible. However, their performance is highly sensitive to the choice of hyperparameters, and determining the appropriate values can be challenging.

Real-world bipartite networks often exhibit heterogeneous node degree distributions, characterized by a few high-degree nodes and many low-degree nodes [18,19]. This imbalance leads to substantial variation in the structure of bipartite networks. However, most existing similarity-based link prediction methods rely primarily on the number of common neighbors or their simple variants, without considering the structural organization of these neighbors. As a result, they are often ineffective at capturing subtle differences in local topology. To address this limitation, we propose the **Nei**ghborhood-enhanced **B**ipartite **L**ink **P**rediction (NeiBLP) method, which not only accounts for the degree heterogeneity of intermediate nodes between unconnected node pairs, but also incorporates their contributions within the local connection patterns they form. This approach enables NeiBLP to differentiate between cases where intermediate nodes have varying degree distributions, an aspect often overlooked by traditional methods. Specifically, the framework leverages quadrangle graphs between unconnected nodes to analyze local topology and introduces two novel metrics to quantify the contributions of cross-type and same-type nodes, facilitating that both structural and degree variations are captured comprehensively.

The main contributions of this paper are summarized as follows:

- Model: The NeiBLP framework introduces a novel, parameter-free similarity approach to tackle degree heterogeneity in bipartite networks. By normalizing the contributions derived from the uv-Quadrangle Graph, the framework effectively mitigates the inherent degree bias commonly observed in such networks.
- Node contribution differentiation: NeiBLP proposes two novel indices, $S^{QDRA}$ and $S^{QSRA}$, to distinguish the contributions of cross-type and same-type nodes. This differentiation effectively accounts for degree effects while simultaneously integrating shared neighbor information.
- Performance: We conducted experiments on ten real-world bipartite networks and compared NeiBLP to nineteen baseline algorithms. Our results demonstrate that NeiBLP outperforms the state-of-the-art bipartite link prediction algorithms and consistently achieves high AUC and Precision scores across diverse bipartite networks.

The rest of this paper is organized as follows. Section 2 provides an overview of related work on link prediction in bipartite networks. In Section 3, we present a detailed description of our proposed method. Section 4 introduces the datasets and their division, baselines, evaluation metrics, and discusses the experimental results. Finally, Section 5 summarizes the findings and concludes the paper.

## 2. Related Work

Recent years have witnessed the emergence of a wide variety of bipartite link prediction algorithms in the literature. These algorithms can be broadly categorized into four groups [2]: similarity-based methods, projection-based methods, dimensionality reduction-based methods, and other methods. In this section, we present a concise overview of representative methods, with a primary focus on similarity-based approaches due to their simplicity, interpretability, and widespread application. Moreover, Table 1 summarizes the advantages, limitations, and key studies associated with each category.

### 2.1. Similarity-Based Methods

Similarity-based methods are among the simplest and most effective approaches for link prediction. Based on their inherent characteristics, these methods can be broadly classified into local, global, and quasi-local indices.

Local indices rely on the immediate neighborhood of target nodes to compute similarity scores. Classic approaches include common neighbor (CN) [20], Adamic–Adar (AA) [21], and resource allocation (RA) [22]. RA leverages degree information of two nodes to compute their similarity, offering a simple yet effective approach for link prediction in unipartite networks. Despite its simplicity, RA has been successfully applied in personalized recommendation and graph reconstruction tasks [23]. Global indices, in contrast, utilize the entire network structure for similarity calculations. For instance, Katz [13] index considers all paths between node pairs, with shorter paths contributing more strongly to the connection probability. Quasi-local methods offer a balance between local and global indices by incorporating both local neighborhood and partial global information. Representative methods include local path (LP) [24] and local random walk [25].

Although these traditional methods are effective in unipartite networks, they encounter limitations when applied to bipartite networks. In bipartite networks, connections exist solely between nodes of two disjoint sets, and no links can form between nodes within the same set. This inherent structural property results in the absence of common neighbors between nodes of two different sets, thereby limiting the applicability of local and quasi-local indices that rely on shared neighbor information.

To address these challenges, researchers have developed modifications to traditional methods and developed new approaches. Cannistraci et al. [9] introduced the LCP theory, which extends the CN concept by considering not only the shared neighbors but also the structural organization of links between these neighbors. Although originally designed for unipartite networks, Daminelli et al. [26] adapted LCP theory for bipartite networks. In this adaptation, common neighbors in bipartite networks are derived from quadratic closures rather than traditional triadic closures. Classical indices, such as AA, CN, and PA, were redefined under this framework using the concept of links between common neighbors. Another promising direction involves methods leveraging the unique structural properties of bipartite networks. Since the shortest path length between two nodes in different sets is three, path-based methods have shown significant potential. For example, Kovács et al. [5] proposed a degree-normalized L3 score, which has proven effective for predicting missing protein–protein interactions in bipartite protein interaction networks. Zhao et al. [27] introduced odd-length path-based link prediction methods, encompassing all three subtypes of similarity-based indices and further expanding their applicability.

### 2.2. Projection-Based Methods

Bipartite networks are a unique network structure, and a common approach for link prediction in such networks is to project the bipartite network onto a unipartite network. Link prediction is then performed in the projected unipartite networks. Unweighted mapping is unable to provide the association strength between nodes of the same type [28,29]. One of the key challenges in projection-based link prediction methods is how to appropriately assign weights to the edges in the projected networks. Zhou et al. [14] proposed a weighting method based on a resource allocation process, where resources are allocated between nodes based on their connections in the original bipartite network. To improve computational efficiency, Gao et al. [15] performed link prediction within candidate node pairs (CNPs) in the projected graph. By focusing on link prediction exclusively within the CNPs, they reduced computational complexity. The connectivity of each CNP is determined by the weights of the patterns it covers. In the context of weighted networks, the concepts of weak and strong links have gained prominence. Aslan et al. [30] proposed the NARM model, which integrates a strengthened projection model with a time-aware proximity measure, allowing for the better capture of temporal dynamics in bipartite networks. Despite these advancements, a key limitation remains: it is difficult to accurately convert a bipartite network into a unipartite network. Moreover, the projected unipartite network must preserve the topological information of the original bipartite network to ensure effective link prediction.

### 2.3. Dimensionality Reduction-Based Methods

Dimensionality reduction-based methods include matrix factorization (MF) and network embedding methods.

Matrix factorization (MF) decomposes the adjacency matrix into the product of multiple low-dimensional matrices to uncover hidden relationships. Pech et al. [31] proposed a robust principal component analysis (RPCA) method, which decomposes the high-dimensional data matrix into a low-rank matrix and an error matrix. Chen et al. [32] focused on capturing intricate high-order relationships between nodes in bipartite networks and integrated these relationships into a unified framework for enhanced prediction accuracy. To capture hierarchical features of bipartite networks, Saberi et al. [33] introduced a deep non-negative matrix factorization method that preserves both global and local structures. The primary difference among them lies in the constraints applied during the factorization process, which influence the extraction of latent factors and the overall quality of the factorization.

Network embedding methods transform networks into low-dimensional vector spaces while preserving the structure information of bipartite network [34,35,36]. A seminal work in this domain is DeepWalk, proposed by Perozzi et al. [37], which pioneered the application of random walks for network embedding and opened a new research direction in the field. For bipartite networks, Huang et al. [38] proposed the BiANE framework, which captures both inter-partition and intra-partition proximities, offering a more comprehensive representation of the bipartite structure. To address the challenge of insufficient negative node pairs, Jing et al. [39] introduced a self-supervised learning method tailored for bipartite graphs. This approach preserves both local inter/intra-type synergies and global co-cluster synergies. However, these methods still face several challenges, including limited interpretability and the need for hyperparameter tuning.

### 2.4. Other Methods

Recent studies have demonstrated the effective application of perturbation theory for link prediction in unipartite networks [40], where it remains one of the most accurate approaches to date [41]. Building on the perturbation theory, Chen et al. [42] extended the perturbation framework to bipartite networks by constructing a two-layer network that integrates both implicit and explicit relationships between nodes. Additionally, Zheng et al. [43] proposed an RNA-disease association prediction model based on structural perturbation method, which effectively identifies biologically significant links within the bipartite networks.

Apart from perturbation-based methods, deep learning approaches have gained considerable attention in link prediction domain. For instance, Salha et al. [44] introduced a model that utilizes linear transformations in both the encoder and decoder, enabling effective processing of graph data. Shin et al. [45] applied a linear graph autoencoder, which facilitates the formation of new links by creating triangles in bipartite graphs. Furthermore, recent research has highlighted the importance of community structure in link prediction. Blöcker et al. [46] proposed MapSim, an information-theoretic measure that assesses node similarities based on the modular compression of network flow. The method is highly interpretable, as the network’s modular structure offers a clear explanation for the observed similarities.

Recently, with the rapid development of graph neural networks (GNNs), capturing network features has become more efficient and effective. Zhang et al. [47] proposed SEAL, a convolutional GNN-based link prediction framework that learns from both latent and explicit features of nodes as well as the structural information of graphs. However, SEAL is primarily designed for homogeneous graphs, whereas many real-world networks exhibit heterogeneous structures. To address this limitation, Zhang et al. [48] generalized SEAL to the bipartite graph link prediction task in recommender systems, introducing the Inductive Graph-based Matrix Completion (IGMC) model. Similar to SEAL, IGMC samples an enclosing subgraph around each target (user, item) pair but adopts a different node labeling scheme tailored for bipartite graphs. To further address the limitations of traditional GNN in modeling unobserved graph structures, Jin et al. [17] proposed a self-supervised learning approach called Self-supervised Reconstructed Graph Learning (SRGL), which simultaneously learns vertex embeddings and reconstructs the graph structure in a mutually beneficial manner.

**Table 1:** Comparison of existing link prediction methods for bipartite networks.

Category	Subcategory	Advantages	Disadvantages	Methods
Similarity-based	Local	Simple and efficient, low computational complexity	Capture limited structure information	CN [26], PA [49], L3 [5], CAR [50]
	Global	Capture global structure information	High computational complexity	LPOP [27], Katz [51]
	Quasi-local	Low time complexity	Limited information, network-dependent	LP35 [27], CNDP [52]
Projection-based	Weighted projection	Advanced unipartite link prediction methods can be used	Loss of bipartite structure information	PLP [15], NBI [14], NARM [30]
	Unweighted mapping	Simple and intuitive	Association strength between nodes of the same type is missing	Refs. [28,29]
Dimensionality reduction-based	Matrix factorization-based	Capture global and local structure	Hyperparameter tunning	DNMF [53], LO [54], BNLP-IEI [32], SRNMF [55]
	Network embedding	Could utilize attribute information for prediction	Hyperparameter tunning, limited interpretability	STERLING [39], BiANE [38]
Other methods	Structural perturbation theory	Efficient and robust	High time complexity	SPM [40], SPRDA [43], SESP [42]
	Information-theoretic	Highly interpretable	High time complexity	PMIL [56], MapSim [46]
	Deep learning	Capture non-linear structure information	Limited interpretability	ICTC [45], LGAE [44]
	GNN-based	Capture complex non-linear structural information	Limited interpretability	SRGL [17], IGMC [48]

## 3. Methodology

This section presents the proposed method, NeiBLP, starting with the problem definition. The limitations of existing structural measures are then discussed to motivate the development of a new approach. Finally, NeiBLP is presented in detail.

### 3.1. Problem Description

Consider an undirected and unweighted bipartite network G(U,V,E), where *U* and *V* are two disjoint sets of nodes, and *E* is the set of edges that connect nodes exclusively between *U* and *V*. The given network G can be represented by an m×n adjacency matrix *B*, where m=|U| and n=|V|. In this matrix, $b_{ij}=1$ if a links exists between two nodes $u_{i}\in U$ and $v_{j}\in V$, and otherwise, $b_{ij}=0$. We further denote all possible m×n edges as the set H, and the set of non-existing edges as H-*E*. The goal of link prediction in bipartite networks is to identify missing edges from the set H-*E*.

### 3.2. From Structural Indistinguishability to a New Index

**Figure 1:**
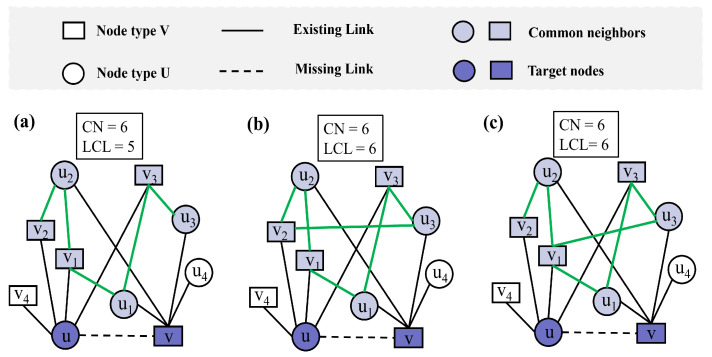
Toy example illustrating common neighbors (CNs) and local community links (LCLs) in bipartite networks. The light purple nodes represent the common neighbors of nodes *u* and *v*, while the green links denote the local community links between common neighbors. (**a**) Nodes *u* and *v* share six common neighbors, resulting in CN = 6 and LCL = 5. (**b**) An additional local community link is added, increasing LCL to 6 while CN remains 6. (**c**) The same CN and LCL values are maintained as in (**b**), but the local topological structure differs due to a change in node degree.

While traditional link prediction metrics provide valuable insights into network structures, they sometimes fail to capture subtle but significant differences in connection patterns. For example, in Figure 1a–c, nodes *u* and node *v* share the same set of common neighbors, i.e., $\left\{u_{1},u_{2},u_{3},v_{1},v_{2},v_{3}\right\}$, where the CN index [26] between nodes *u* and *v* is consistently 6. Thus, using CN alone cannot differentiate the link likelihood between these node pairs. Although the overall network topology remains largely consistent across the three subfigures, subtle changes in the local connections among common neighbors lead to differences in the LCL values. Specifically, compared with Figure 1a,b, which form an additional link, the LCL is increased to 6; and Figure 1c maintains the same LCL value as (b) but with a different pattern of interconnections. This highlights the importance of considering the connectivity between the neighbors of the two unconnected nodes, as it may vary despite identical sets of common neighbors.

Although the CN index and LCL index [50] between nodes *u* and *v* remain the same across Figure 1b,c, differences in the local topological structure are evident. For instance, in Figure 1b, the degree of $v_{1}$ is 3, while in Figure 1c, the degree of $v_{1}$ is 4. This example illustrates that the LCL index fails to account for such variations in node degrees within the local topology, which can influence link prediction performance. To address these shortcomings, we propose a new index that better captures local structural variations in bipartite networks.

### 3.3. NeiBLP: The Proposed Framework

In this section, we present two preliminary definitions that will be used in the rest of this paper. The concepts are defined as follows.

**Definition** **1** (uv-Quadrangle Graph)**.** *Given two nodes u∈U and v∈V, the uv-Quadrangle Graph, denoted as $Q_{uv}$, is the subgraph of the original bipartite network G, consisting of all nodes and edges that belong to length-three (L3) paths between u and v, including u and v themselves.*

**Definition** **2** (Same-type resource allocation in bipartite networks)**.** *Inspired by the resource allocation (RA) index for unipartite networks, the same-type resource allocation (STRA) index extends the RA index to bipartite networks, specifically focusing on computing RA scores between nodes of the same type. Formally, the STRA score between two nodes u∈U and t∈U is defined as*

(1) $$S_{ut}^{STRA}=\sum_{x\in \Gamma (u)\cap \Gamma (t)}\frac{1}{k_{x}}$$

*where $k_{x}$ denotes the degrees of node x, and Γ(u) and Γ(t) represent the sets of all neighbors of u and t, respectively. The STRA index captures the intuitive idea that two nodes are more likely to connect if they share neighbors with low degrees.*

**Figure 2:**
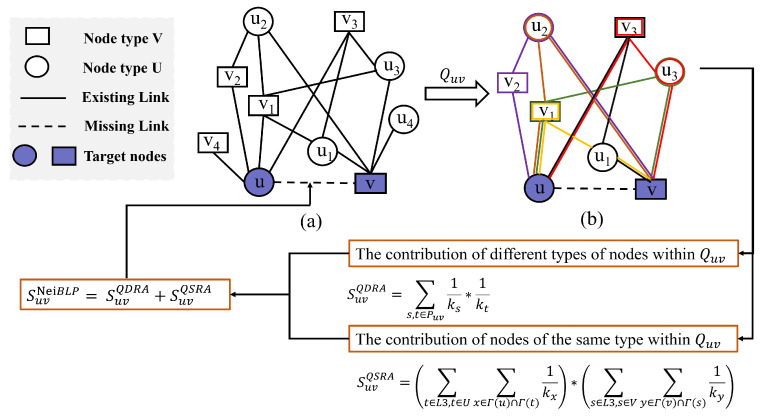
The flowchart of NeiBLP. (**a**) An example of a bipartite network with target nodes *u* and *v*. (**b**) The extracted uv-Quadrangle Graph from (**a**). The different colors represent different L3 paths in $Q_{uv}$.

Existing neighborhood-based indices in bipartite networks often neglect local connectivity patterns. To address the limitations, we propose the **Nei**ghborhood-enhanced **B**ipartite **L**ink **P**rediction (NeiBLP) method. NeiBLP not only considers the degree heterogeneity of intermediate nodes between unconnected node pairs, but also incorporates the contributions of these nodes within the local connection patterns they form. NeiBLP can differentiate between such cases because the intermediate nodes have different degree distributions, which are often overlooked by traditional methods. Furthermore, NeiBLP preserves the neighborhood interaction structure between unconnected nodes in bipartite networks. The flowchart of the NeiBLP is shown in Figure 2.

Due to the effectiveness of the RA in unipartite networks, we extend it to bipartite networks by redefining the contributions of cross-type nodes within the framework established in Definition 1. This extension enables RA to better account for the structural characteristics unique to bipartite networks.

For a pair of target nodes u∈U and v∈V representing a potential interaction, let $Q_{uv}$ denote the uv-Quadrangle Graph as defined in Definition 1. The $Q_{uv}$-based resource allocation (QDRA) for *u* and *v* is defined as

(2) $$S_{uv}^{QDRA}=\sum_{\left(s,t\right)\in P_{uv}}\frac{1}{k_{s}}\cdot \frac{1}{k_{t}}$$

where $P_{uv}$ is the set of all distinct L3 paths within $Q_{uv}$, and $k_{s}$ and $k_{t}$ denote the degrees of intermediate nodes s∈V and t∈U, respectively. This normalization mitigates the influence of degree bias caused by variations in the intermediate nodes.

To better understand the structural characteristics of bipartite networks, we consider four real-world datasets: GPC, Ion, Malaria, and Drug. Each dataset consists of two disjoint types of nodes (denoted as U and V), with edges representing observed associations between them. These datasets are further described in Section 4.1.

**Figure 3:**
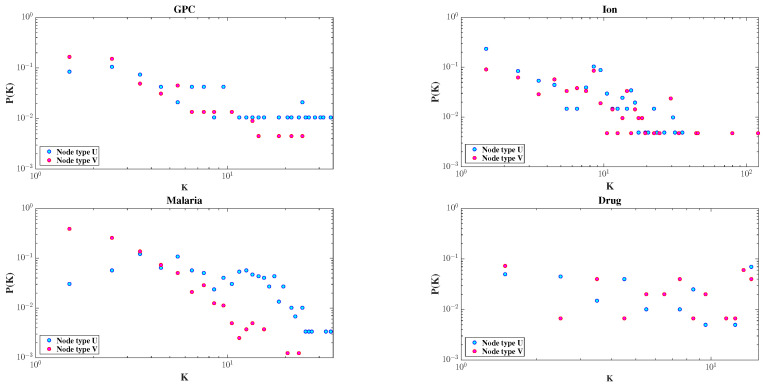
Degree distributions of node types U and V in the GPC, Ion, Malaria, and Drug datasets, respectively, plotted on a double logarithmic scale.

The degree distributions of the two node types are shown in Figure 3. All four datasets exhibit skewed distributions; most nodes have very few connections, while a few serve as hubs. This highlights the heterogeneous nature of bipartite networks.

In such networks, node interactions are often influenced by biases originating from both same-type and cross-type neighbors. These biases can vary noticeably across different scenarios, thereby affecting the structural relationships between nodes. To effectively model this heterogeneity, it is important to account for the contributions of same-type nodes, under the assumption that nodes sharing common neighbors are more likely to exhibit higher similarity.

The same-type contribution, denoted as $S_{uv}^{QSRA}$, captures the incremental similarity score contributed by same-type nodes along $Q_{uv}$ and is defined as

(3) $$S_{uv}^{QSRA}=\left(\sum_{t\in L3,t\in U}\sum_{x\in \Gamma (u)\cap \Gamma (t)}\frac{1}{k_{x}}\right)\times \left(\sum_{s\in L3,s\in V}\sum_{y\in \Gamma (v)\cap \Gamma (s)}\frac{1}{k_{y}}\right)$$

Here, t∈U and s∈V denote same-type intermediate nodes of *u* and *v*, respectively. The sets Γ(u), Γ(v), Γ(t), and Γ(s) represent the neighbors of nodes *u*, *v*, *t*, and *s*, while $k_{x}$ and $k_{y}$ indicate the degrees of nodes x∈V and y∈U, respectively.

Due to considerations of complementarity, $S_{uv}^{QDRA}$ and $S_{uv}^{QSRA}$ capture different aspects of structural information. Addition allows these complementary informations to jointly influence the final similarity score, ensuring that both types of structural characteristics are effectively taken into account. The combined similarity score $S_{uv}^{NeiBLP}$ is given by

(4) $$S_{uv}^{NeiBLP}=S_{uv}^{QDRA}+S_{uv}^{QSRA}$$

To illustrate the proposed NeiBLP framework, Figure 2 provides a step-by-step example. Specifically, Figure 2a depicts the original bipartite network, while Figure 2b shows a subgraph, denoted as $Q_{uv}$, which contains six distinct L3 paths between nodes *u* and *v*. For example, one such path is $u-v_{1}-u_{1}-v$. Within the $Q_{uv}$ subgraph, the NeiBLP framework separately calculates contributions by distinguishing the roles of same-type and cross-type nodes. For the cross-type contribution, the NeiBLP framework computes $S_{uv}^{QDRA}=\frac{1}{k_{v_{1}}}\cdot \frac{1}{k_{u_{1}}}=\frac{1}{12}$. For the same-type contribution, $S_{uv}^{QSRA}$ is calculated as $S_{uv}^{QSRA}=S_{uu_{1}}^{STRA}+S_{vv_{1}}^{STRA}=\left(\frac{1}{k_{v_{1}}}+\frac{1}{k_{v_{3}}}\right)\cdot \left(\frac{1}{k_{u_{1}}}+\frac{1}{k_{u_{2}}}+\frac{1}{k_{u_{3}}}\right)=\frac{7}{12}$. This example demonstrates how the NeiBLP framework effectively incorporates both same-type and cross-type node contributions by accounting for the degree heterogeneity of nodes within the local structure $Q_{uv}$.

### 3.4. Algorithm Description

The NeiBLP framework differentiates between the roles of same-type nodes (nodes of the same type) and cross-type nodes (nodes of different types) by leveraging all L3 paths within the $Q_{uv}$ between two unconnected nodes. The calculation process is provided in Algorithm 1.
**Algorithm 1** The calculation process of NeiBLP framework**Input:** Bipartite network G=(U,V,E). **Output:** Predicted similarity matrix $S^{NeiBLP}$.
  1:**begin**  2:**for** m=1 **to** 100 **do**  3:   Divide *B* into training set $B^{T}$ and testing set $B^{P}$  4:   Calculate the contribution of cross-type nodes along  $Q_{uv}$ according to Equation (2)  5:   $S_{uv}^{QDRA}\leftarrow \sum_{\left(s,t\right)\in P_{uv}}\frac{1}{k_{s}}\cdot \frac{1}{k_{t}}$  6:   Calculate the contribution of same-type nodes along  $Q_{uv}$ according to Equation (3)  7:   $S_{uv}^{BSRA}\leftarrow \left(\sum_{t\in L3,t\in U}\sum_{x\in \Gamma (u)\cap \Gamma (t)}\frac{1}{k_{x}}\right)\left(\sum_{s\in L3,s\in V}\sum_{y\in \Gamma (v)\cap \Gamma (s)}\frac{1}{k_{y}}\right)$  8:   Update $S_{uv}^{NeiBLP}$ according to Equation (4)  9:   $S_{uv}^{NeiBLP}\leftarrow S_{uv}^{QDRA}+S_{uv}^{QSRA}$10:**end for**11:**end**


### 3.5. Complexity Analysis

The $S_{uv}^{NeiBLP}$ consists of two parts: $S_{uv}^{QDRA}$ and $S_{uv}^{QSRA}$. For a node pair (u,v)∈U×V, computing $S_{uv}^{QDRA}$ involves enumerating all distinct length-3 paths from *u* to *v* within the $Q_{uv}$ quadrangle graph. Since each step involves visiting nodes with an average degree *d*, this process has a time complexity of $O(d^{2})$. Similarly, calculating $S_{uv}^{QSRA}$ requires finding common neighbors between *u* and its same-type intermediate nodes, and between *v* and its same-type intermediate nodes, each contributing an additional $O(d^{2})$ time. Thus, the overall time complexity for computing $S_{uv}^{NeiBLP}$ for a node pair is $O(d^{2})$ In the worst-case scenario where all possible |U|×|V| node pairs are considered for link prediction, the overall time complexity is $O(|U|\times |V|\times d^{2})$.

## 4. Experimental Results

In this section, we evaluate the effectiveness of our proposed NeiBLP method using ten real-world bipartite networks. The experimental results demonstrate a consistent improvement in performance achieved by our method.

### 4.1. Datasets

To evaluate the performance of different algorithms, we utilized ten real-world bipartite networks from various domains as datasets. In these networks, $\left|U\right|$ and $\left|V\right|$ represent the number of nodes in the two distinct sets, while $\left|E\right|$ denotes the total number of edges. Key properties include average degree of nodes in *U* ($\langle k_{U}\rangle$), and average degree of nodes in *V* ($\langle k_{V}\rangle$). Sparsity indicates the proportion of unobserved interactions relative to the maximum possible number of interactions. The statistical characteristics of these datasets are summarized in Table 2.

The specific description of these networks are follows: (a) G-protein coupled receptors (GPC) [57]: This biological bipartite network consists of 223 drugs, 95 target proteins, and 635 experimentally validated drug–target interaction pairs. (b) Enzymes [57]: This biological bipartite network includes 445 drugs, 664 target proteins, and 2926 experimentally verified drug–target interaction pairs. (c) Ion channels (Ion) [57]: This biological bipartite network comprises 210 drugs, 204 target proteins, and 1476 experimentally confirmed drug–target interaction pairs. (d) Malaria [58]: This genetic bipartite network represents genetic sequences from the malaria parasite plasmodium falciparum. It includes 297 genes and 806 shared amino acid subsequences. (e) Drug–target (Drug) [59]: This biological bipartite network consists of 200 drugs, 150 target proteins, and 454 experimentally validated drug–target interaction pairs. (f) Southern women (SW) [60]: This social bipartite network represents 89 interactions between 18 white women and 14 social events. An edge exists between a woman and an event if she participated in that event. (g) Country–organization (C2O) [61]: This global bipartite network consists of 144 country nodes and 155 organization nodes, connected through 12,170 affiliation links. Each link represents a country’s membership or participation in an international organization. (h) Na-net [62]: This air transportation bipartite network consists of 940 city nodes and 940 coordinate nodes, connected by 6892 links. An edge indicates that a city is associated with a specific coordinate node based on the geographical position of its airport. (i) MovieLens100K (ML100K) (https://www.grouplens.org (accessed on 23 May 2025)): This social bipartite network consists of 943 users and 1574 movies, with a total of 82,520 user–movie rating interactions. The ratings range from 1 to 5, and in our experiments, we consider a link to exist between a user and a movie if the rating is greater than or equal to 3. (j) DBLP (https://www.dblp.uni-trier.de/xml (accessed on 23 May 2025)): This publication bipartite network represents the publishing relationships between 6001 authors and 1308 venues. An edge exists between an author and a venue if the author has published a paper at that venue.

### 4.2. Division of Datasets

To validate the accuracy of link prediction algorithms, bipartite network datasets are divided into training set and testing set based on different partition ratios. In this process, the set of edges removed for testing is denoted as $E^{P}$, while the remaining edges constitute the training set, represented as $E^{T}$. By definition, $E^{T}\cup E^{P}=E$ and $E^{T}\cap E^{P}=Ø$. The algorithm’s predictive performance is assessed by its ability to identify edges in the testing set $E^{P}$. Specifically, the prediction results are ranked in descending order, where edges from $E^{P}$ occupy the highest ranks and edges from H−E appear at lower ranks.

### 4.3. Baseline Algorithms

In this paper, we select a total of nineteen representative link prediction methods from eight different categories in bipartite networks as baselines. These include three neighborhood-based methods, four path-based methods, two projection-based methods, three LCP-based methods, one structural perturbation method, four dimensionality reduction-based methods, one deep learning method, and one mutual information-based method. The selected baseline methods are summarized in Table 3.

### 4.4. Evaluation Metrics

Precision and area under the receiver operating characteristic curve (AUC) are used to measure the performance of various link prediction methods. Precision specifically measures the proportion of correctly predicted links, while AUC provides a comprehensive evaluation of the model’s overall performance.

(1) Precision

Precision [64] evaluates the effectiveness of a link prediction model in correctly identifying relevant or true positive links among its predictions. Specifically, let the set of potential links be denoted as $E^{P}$. If we rank all potential links by their similarity scores in descending order and select the top *L* links as the predicted missing links, and let $L_{r}$ be the number of these that are correctly predicted, then Precision is given by

(5) $$Precision=\frac{L_{r}}{L}$$

In practice, *L* is often set to equal to the number of links in the test set $|E^{P}|$.

(2) AUC

Compared to Precision, AUC [65] measures the probability that a missing link receives a higher score than a non-existent link. For instance, consider *n* independent comparisons, where in each comparison, a missing link and a non-existent link are randomly selected to compare their scores. If the missing link has a higher score in $n^{\prime }$ cases and both links have the same score in $n^{\prime \prime }$ cases, the AUC value is calculated as follows:

(6) $$AUC=\frac{n^{\prime }+0.5n^{\prime \prime }}{n}$$

Obviously, higher scores in Equations (5) and (6) indicate greater prediction accuracy.

To evaluate prediction accuracy, the observed links *E* are randomly divided into a training set $E^{T}$ and a test set $E^{P}$. All reported results are averaged over 100 independent runs. In our evaluation, we compute scores for all non-observed links between nodes in the bipartite network. Specifically, for each test link in $E^{P}$, its score is compared against those of all other node pairs that are not connected in the training set $E^{T}$. This setting ensures a more comprehensive and rigorous evaluation, particularly suitable for sparse bipartite networks.

### 4.5. Experiment Analysis

In this section, we present three experiments that were conducted to evaluate the performance of our proposed NeiBLP. First, we assessed its overall performance to evaluate the method’s effectiveness. Second, we analyzed its robustness under different training set ratios. Third, we performed ablation studies to investigate the contribution and effectiveness of each component within the framework.

#### 4.5.1. Comparison with Baselines

To evaluate the performance of NeiBLP, we used AUC and Precision as the evaluation metrics for predicting missing links. For each experimental network, we partitioned 90% of the links as the training set, while the remaining 10% links were used as the testing set. To ensure robust results, we conducted 100 independent experiments for each method and calculated the average AUC and Precision. The results are summarized in Table 4 and Table 5, with the best values highlighted in bold and the second-best underlined. The hyperparameters used for the baseline methods are listed in Table 6.

In terms of AUC, NeiBLP achieves either the best or the second-best performance, showing consistent improvement over other baseline methods.

Several baseline methods could not be fully evaluated in our experimental comparison due to technical limitations. Specifically, as the PMIL method lacked publicly available source code, reimplementation was conducted based on the descriptions provided in the original publication. In addition, the ICTC was unable to operate effectively on the drug network, as its transformation into single-mode sparse matrices resulted in substantial information loss in highly sparse networks. Consequently, the corresponding entries in Table 4 and Table 5 are marked with the symbol “−” to denote unavailable results. Overall, perturbation-based method exhibits better predictive performance than neighborhood-based and LCP-based approaches. Among the path-based methods (i.e., L3, LP3, LP35, and LPOP), LP35 and LPOP consistently perform worse than LP3 across all datasets, highlighting the ineffectiveness of using paths longer than three. L3 achieves the best performance because it normalizes node degrees, mitigating the bias introduced by the varying degrees of intermediate nodes.

In terms of Precision, the LP3 exhibits lower performance than L3 across most bipartite networks. This observation suggests that solely considering third-order paths is insufficient to capture the connectivity between nodes, highlighting the necessity of considering node biases. LPOP shows a slight improvement over LP35 but remains inferior to L3. These results suggest that incorporating higher-order paths (e.g., LP35 and LPOP) often introduces additional invalid paths, which increases prediction noise and ultimately degrades performance. For neighborhood-based approaches, these methods generally demonstrate stable performance across most networks. RA typically outperforms CN and AA, which may be attributed to the fact that, in addition to considering common neighbors, RA considers the degree heterogeneity of the common neighbors. Among LCP-based approaches, CRA achieves the best performance, outperforming CAR and CAA on most bipartite networks, particularly on datasets such as Enzymes, SW, and ML. The SESP method, which employs a structural perturbation strategy and incorporates both implicit and explicit relationships, demonstrates strong performance on dense networks such as C2O, Na-net, and ML100K. However, its computational complexity is relatively high, which may limit its scalability in certain scenarios. Dimensionality reduction-based approaches exhibit strong performance on specific networks; however, these methods often involve a large number of hyperparameters, making their application more complex and less practical. In contrast, the NeiBLP method consistently achieves optimal performance across ten bipartite networks, demonstrating its stability and superior effectiveness.

#### 4.5.2. Robustness Analysis

To evaluate the robustness of NeiBLP, we set the proportion of the training set *p* to 0.4, 0.6, and 0.8, and investigated the Precision and AUC under different training set ratios. A lower ratio indicates that more links are removed as the testing set. To ensure sufficient training set, we explored training set ratios no less than 0.4.

**Figure 4:**
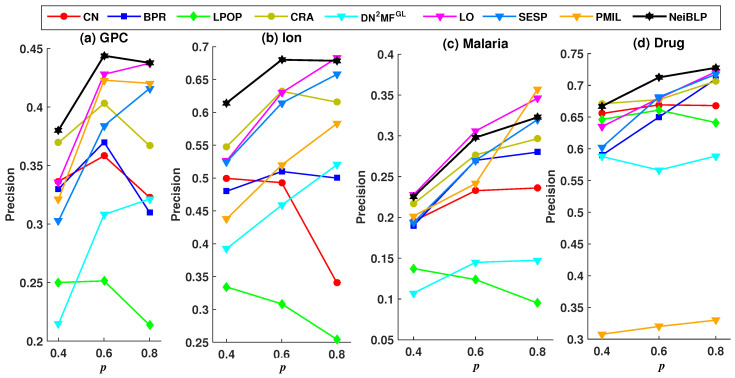
Precision results under different training set ratios *p*.

**Figure 5:**
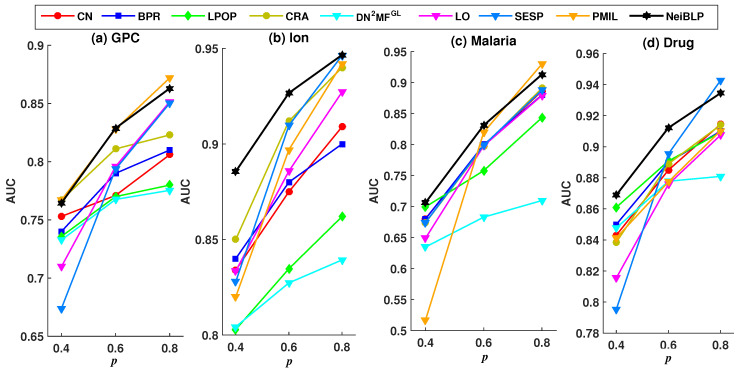
AUC results under different training set ratios *p*.

Considering the readability of the figures, we selected eight representative and relatively strong baseline methods for comparison. The evaluation results under different training set ratios are shown in Figure 4 and Figure 5.

In Figure 4 and Figure 5, the black line represents the performance of our NeiBLP, while the other color lines correspond to various approaches: red for neighborhood-based methods, yellow for LCP-based methods, dark blue for projection-based methods, green for path-based methods, light blue for structural perturbation, pink for dimensionality reduction, and orange for the mutual information-based approach. From Figure 4, when the proportion of the training set decreases from 0.8 to 0.4, the proportion of missing links increases. The performance of NeiBLP exceeds that of other baseline methods, suggesting that NeiBLP is more effective under conditions with limited observable link information. This is particularly significant, as real-world bipartite networks are usually sparse. Additionally, an interesting trend is observed, for neighborhood-based and LCP-based methods, the Precision improves as the training set ratio increases from 0.4 to 0.6. However, when the training set ratio further increases from 0.6 to 0.8, a decline in Precision is observed.

As demonstrated in Figure 5, with the increases in the proportion of the training set *p*, all methods exhibit a consistent upward trend. The NeiBLP demonstrates the most stable performance across the four datasets and consistently achieves either the best or second-best results. As shown in Table 4, when the training data ratio reaches 0.9, the AUC and Precision values for the GPC and Malaria datasets in the PMIL method are lower than those of NeiBLP. Overall, the NeiBLP curve almost lies above the curves of other baseline methods, indicating that our method demonstrates superior robustness compared to others under different training set ratios.

#### 4.5.3. Ablation Study

**Figure 6:**
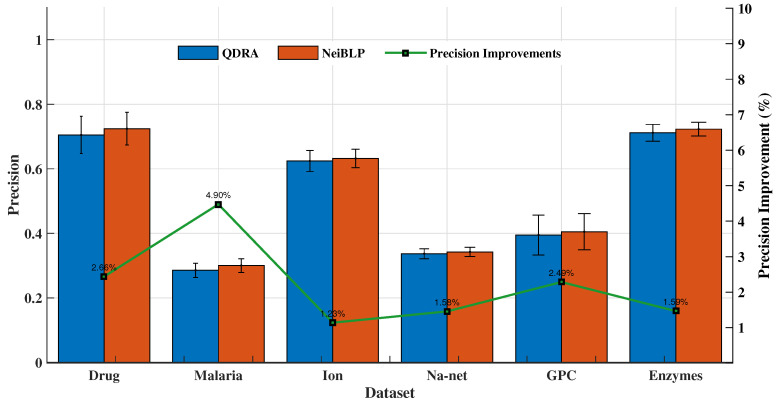
Link prediction results of ablation experiments, QDRA vs. NeiBLP. The error bars represent the standard deviation.

**Figure 7:**
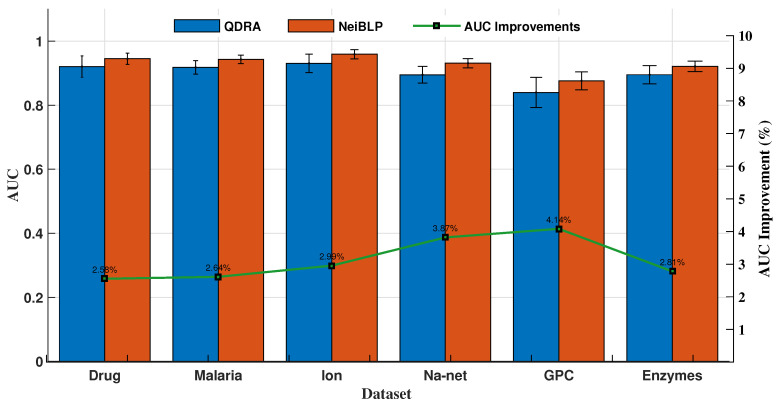
Link prediction results of ablation experiments, QDRA vs. NeiBLP. The error bars represent the standard deviation.

The QDRA index considers the contributions of different types of nodes within $Q_{uv}$ in bipartite networks, while NeiBLP extends beyond QDRA by also considering the contributions of nodes of the same type within $Q_{uv}$. We evaluated the performance of QDRA and NeiBLP using AUC and Precision. Detailed comparisons are provided in Figure 6 and Figure 7. As shown in the figures, NeiBLP outperforms QDRA across all experimental datasets. The Precision improvements of NeiBLP over QDRA range from 1.23% to 4.90%, while the AUC improvements range from 2.58% to 4.14%. Moreover, NeiBLP demonstrates lower variance in performance compared to QDRA, highlighting its superior stability. The advantage of NeiBLP lies in its ability to consider the similarities among nodes of the same type within $Q_{uv}$.

## 5. Conclusions and Discussion

In real-world bipartite networks, heterogeneous node degree distributions often undermine the effectiveness of traditional local structure-based link prediction methods, particularly in capturing subtle topological differences. To address this limitation, we propose NeiBLP, a parameter-free and interpretable link prediction framework that explicitly accounts for the degree heterogeneity of intermediate nodes between unconnected node pairs. By incorporating the influence of these intermediate nodes within local connection patterns, NeiBLP helps to improve prediction accuracy.

Unlike traditional methods, which are interpretable by nature but lack flexibility in handling structural heterogeneity, NeiBLP’s decomposition into cross-type resource allocation and same-type reinforcement components offers a more intuitive and fine-grained understanding of the underlying structure of predicted links. Comprehensive experiments on ten diverse real-world bipartite networks demonstrate that NeiBLP consistently achieves the best or second-best performance compared to nineteen state-of-the-art link prediction methods, confirming its effectiveness and robustness. In future work, the flexible design of NeiBLP can be further leveraged to incorporate node attribute information, where attribute similarities between same-type nodes could enrich the modeling of complex relationships. Moreover, enhancing the scalability of NeiBLP through the development of parallel implementations presents a promising direction to improve computational efficiency and broaden its applicability to large-scale bipartite networks.

## Figures and Tables

**Table 2:** Basic topological properties of ten bipartite networks.

Network	$\left|U\right|$	$\left|V\right|$	$\left|E\right|$	$\langle k_{U}\rangle$	$\langle k_{V}\rangle$	Sparsity (%)
GPC	95	223	635	6.68	2.85	97.00
Enzymes	664	445	2926	4.41	6.58	99.01
Ion	210	204	1476	7.03	7.24	96.55
Malaria	297	806	2965	9.98	3.68	98.76
Drug	200	150	454	2.27	3.03	98.49
SW	18	14	89	4.94	6.36	64.68
C2O	144	151	12,170	84.51	80.60	44.03
Na-net	940	940	6892	12.95	12.95	99.22
ML100K	1574	943	82,520	52.43	87.51	94.42
DBLP	6001	1308	29,256	4.88	22.37	99.63

**Table 3:** Baseline methods for link prediction.

Method	Formula	Parameter Description
CN [26]	$S_{uv}^{CN}=\left|\left(\Gamma \left(u\right)\cap \hat{\Gamma }\left(v\right)\right)\cup \left(\hat{\Gamma }\left(u\right)\cap \Gamma \left(v\right)\right)\right|$	$\hat{\Gamma }\left(u\right)=\cup_{s\in \Gamma (u)}\Gamma \left(s\right)$ Γ(u) represents the set of neighbors of *u*
JC [26]	$S_{uv}^{JC}=\frac{S_{uv}^{CN}}{\left|\Gamma (u)\cup \Gamma (v)\right|}$	
AA [26]	$S_{uv}^{AA}=\sum_{z\in \left(\Gamma \left(u\right)\cap \hat{\Gamma }\left(v\right)\right)\cup \left(\hat{\Gamma }\left(u\right)\cap \Gamma \left(v\right)\right)}\frac{1}{\left|\Gamma (z)\right|}$	
LP3 [27]	$S_{uv}^{LP3}={\left(B^{3}\right)}_{uv}$	*B* denotes the adjacency matrix, $B^{3}$ denotes third-order paths
LP35 [27]	$S_{uv}^{LP35}={\left(B^{3}\right)}_{uv}+\beta {\left(B^{5}\right)}_{uv}$	β is a hyperparameter used to control the contribution of the third-order paths, $B^{5}$ denotes fifth-order paths
L3 [5]	$S_{uv}^{L3}=\sum_{s,t}\frac{b_{us}b_{st}b_{tv}}{\sqrt{\left|\Gamma (u)||\Gamma (v)\right|}}$	$b_{us}$ denotes whether there is an interaction between nodes *u* and *s*. If such interaction exists, then $b_{us}=1$, otherwise $b_{us}=0$
LPOP [27]	$S_{uv}^{LPOP}={\left(\beta B+\beta^{3}B^{3}+\beta^{5}B^{5}+\cdots \right)}_{uv}$	β is a hyperparameter that controls the weight of different odd-length paths
CAR [50]	$S_{uv}^{CAR}=S_{uv}^{CN}\times S_{uv}^{LCL}$	$S_{uv}^{LCL}=\left|\left\{(s,t):(s,t)\in E,t\in \Gamma (v),s\in \Gamma (u)\right\}\right|$
CAA [50]	$S_{uv}^{CAA}=\sum_{z\in \left(\Gamma \left(u\right)\cap \hat{\Gamma }\left(v\right)\right)\cup \left(\hat{\Gamma }\left(u\right)\cap \Gamma \left(v\right)\right)}\frac{\left|\gamma (z)\right|}{log_{2}\left|\Gamma \left(z\right)\right|}$	$\hat{\Gamma }\left(u\right)=\cup_{s\in \Gamma (u)}\Gamma \left(s\right)$
CRA [50]	$S_{uv}^{CRA}=\sum_{z\in \left(\Gamma \left(u\right)\cap \hat{\Gamma }\left(v\right)\right)\cup \left(\hat{\Gamma }\left(u\right)\cap \Gamma \left(v\right)\right)}\frac{\left|\gamma (z)\right|}{\left|\Gamma (z)\right|}$	$\hat{\Gamma }\left(u\right)=\cup_{s\in \Gamma (u)}\Gamma \left(s\right)$
NBI [14]	$f^{\prime }\left(x_{i}\right)=\sum_{j=1}^{n}\omega_{ij}f\left(x_{j}\right)$	$w_{ij}=\frac{1}{k(x_{j})}\sum_{l=1}^{m}\frac{a_{il}a_{jl}}{k(y_{l})}$
BPR [63]	$f^{\prime }\left(a_{i}\right)=\sum_{j=1}^{\left|A\right|}s\left(a_{i},a_{j}\right)f\left(a_{j}\right)$	$s\left(a_{i},a_{j}\right)=\frac{1}{k(a_{j})}\sum_{l=1}^{\left|B\right|}\frac{N_{il}N_{jl}}{k(b_{l})}$
SESP [42]	${\tilde{B}}^{E}=\sum_{k=1}^{n}\left(\lambda_{k}+\Delta \lambda_{k}\right)x_{k}x_{k}^{T}$	$\lambda_{k}$ and $x_{k}$ correspond to the k-th eigenvalue and eigenvector
SRNMF [55]	$\begin{array}{c} \min\;O\left(x,y\right)=\frac{1}{2}\sum_{i=1}^{n}\sum_{j=1}^{m}{\left(A_{ij}-\sum_{k=1}^{K}x_{ik}\cdot y_{kj}\right)}^{2}+\\ \frac{1}{2}\gamma \sum_{i=1}^{n}\sum_{j=1}^{m}{\left(A_{ij}-\sum_{k=1}^{K}x_{ik}\cdot y_{kj}\right)}^{2}\cdot S_{ij}+\\ \frac{1}{2}\lambda \left(\sum_{i}\sum_{p}x_{ip}^{2}\right)+\frac{1}{2}\lambda \left(\sum_{j}\sum_{q}y_{qj}^{2}\right) \end{array}$	λ and γ are the balance parameters, $S_{ij}$ denotes the similarity between nodes *i* and *j*
RPCA [31]	$\min_{X^{*},E}{∥A-E∥}_{*}+\lambda {∥E∥}_{1}$	the weight parameter
D$n^{2}MF^{GL}$ [33]	a deep non-negative matrix factorization method with joint global and local structure preservation	the number of layers, the size of each layer, the balancing parameters
LO [54]	$E={\alpha ∥A-\mathrm{AZ}∥}_{F}^{2}+{∥Z∥}_{F}^{2}$	α is a free parameter that balances the two requirements
ICTC [45]	ICTC leverages a linear graph autoencoder (LGAE) to capture intra-class relationships	learning rate, hidden dimension, and epoch
PMIL [56]	$PMIS\left(A,i\right)=\sum_{\left\{A,B\right\}\in \Gamma \left(A,i\right)}W\left(A,B\right)$	W(A,B) is the weight of pattern $\left\{A,B\right\}$

**Table 4:** The AUC Results on ten networks. The best values are highlighted in bold, and the second-best are underlined.

	Drug	Malaria	Ion	Na-Net	C2O	GPC	ML100K	SW	Enzymes	DBLP
CN	0.917	0.898	0.920	0.875	0.991	0.814	0.873	0.731	0.853	0.850
AA	0.925	0.909	0.934	0.898	0.988	0.848	0.878	0.723	0.869	0.890
RA	0.930	0.917	0.928	0.901	1.000	0.840	0.888	0.766	0.857	0.767
CAR	0.904	0.906	0.916	0.832	0.990	0.796	0.912	0.727	0.867	0.883
CAA	0.906	0.923	0.922	0.891	1.000	0.830	0.910	0.759	0.853	0.833
CRA	0.928	0.906	0.936	0.864	1.000	0.820	0.920	0.777	0.892	0.850
L3	0.916	0.917	0.926	0.884	$\underline{0.998}$	0.831	0.911	0.777	0.893	0.860
LP3	0.909	0.904	0.915	0.873	0.991	0.825	0.902	0.738	0.894	0.850
LP35	0.912	0.886	0.869	0.837	0.991	0.800	0.880	0.703	0.880	0.803
LPOP	0.911	0.876	0.857	0.836	0.990	0.785	0.886	0.663	0.850	0.838
SESP	0.947	0.919	$\underline{0.958}$	0.858	0.996	0.862	0.893	0.796	0.955	0.850
NBI	0.911	0.921	0.925	0.910	0.997	0.831	0.910	0.764	0.898	0.880
BPR	0.917	0.904	0.913	0.883	$\underline{0.998}$	0.841	0.900	0.740	0.888	$\underline{0.885}$
SRNMF	0.916	0.932	0.929	0.908	1.000	0.820	0.934	0.807	0.887	0.893
D$n^{2}MF^{GL}$	0.901	0.704	0.845	0.788	0.996	0.786	0.892	0.742	0.805	0.798
LO	0.904	$\underline{0.935}$	0.938	0.879	0.996	0.845	0.802	0.778	0.894	0.780
RPCA	0.868	0.596	0.796	0.507	0.580	0.518	0.501	0.572	0.653	0.555
ICTC	−	0.870	0.930	0.960	0.990	0.970	0.920	0.839	0.962	0.560
PMIL	$\underline{0.945}$	0.921	0.938	−	0.971	0.867	$\underline{0.945}$	0.945	0.901	−
NeiBLP	$\underline{0.945}$	0.943	0.959	$\underline{0.931}$	1.000	$\underline{0.876}$	0.946	$\underline{0.878}$	$\underline{0.921}$	0.910

**Table 5:** The Precision results on ten networks. The best values are highlighted in bold, and the second-best are underlined.

	Drug	Malaria	Ion	Na-Net	C2O	GPC	ML100K	SW	Enzymes	DBLP
CN	0.608	0.188	0.190	0.289	0.873	0.306	0.138	0.144	0.374	0.000
AA	0.643	0.215	0.213	0.295	0.873	0.346	0.132	0.167	0.294	0.000
RA	0.693	0.221	0.212	0.297	0.885	0.330	0.103	0.178	0.293	0.000
CAR	0.597	0.187	0.432	0.218	0.873	0.290	0.177	0.189	0.507	0.000
CAA	0.591	0.189	0.537	0.325	0.873	0.359	0.191	0.122	0.503	0.000
CRA	0.630	0.253	0.548	0.305	0.878	0.361	0.184	0.211	0.641	0.000
L3	0.645	0.264	0.601	0.323	0.879	0.325	0.183	0.196	0.526	$\underline{0.114}$
LP3	0.610	0.183	0.480	0.299	0.872	0.272	0.182	0.157	0.490	0.093
LP35	0.570	0.109	0.260	0.275	0.874	0.192	0.171	0.149	0.475	0.076
LPOP	0.595	0.066	0.217	0.275	0.875	0.181	0.173	0.120	0.461	0.057
SESP	0.694	0.299	0.645	0.345	0.931	0.394	0.258	0.173	0.716	0.086
NBI	0.686	0.250	0.593	$\underline{0.342}$	0.908	0.361	0.184	0.194	0.616	0.113
BPR	0.684	0.240	0.437	0.289	0.894	0.271	0.185	0.163	0.495	0.076
SRNMF	0.701	0.259	$\underline{0.678}$	0.335	0.904	0.413	0.190	0.192	0.700	0.041
D$n^{2}MF^{GL}$	0.626	0.136	0.542	0.253	0.906	0.300	0.189	0.178	0.564	0.044
LO	0.696	0.341	0.707	0.208	0.905	0.401	0.081	0.155	$\underline{0.720}$	0.033
RPCA	$\underline{0.717}$	0.174	0.554	0.005	0.596	0.177	0.001	0.148	0.467	0.001
ICTC	−	0.122	0.279	0.276	0.906	0.286	0.180	0.012	0.175	0.003
PMIL	0.310	0.261	0.581	−	0.601	0.401	$\underline{0.210}$	0.341	0.661	−
NeiBLP	0.724	$\underline{0.300}$	0.632	0.345	$\underline{0.912}$	$\underline{0.405}$	0.185	$\underline{0.256}$	0.723	0.120

**Table 6:** Hyperparameters for the baseline methods. $\lambda_{max}$ represents the maximum eigenvalue of the matrix.

Methods	Parameters	All Datasets
SRNMF	regularization parameter	2
	balance parameter	0.5
	cumulative contribution rate	0.95
D$n^{2}MF^{GL}$	latent space	80–10
	balancing parameter α	1
	balancing parameter β	1
	balancing parameter γ	1
ICTC	learning rate	0.1
	hidden dimension	32
	epoch	200
SESP	perturbation rate	0.9
LP35	weight of fifth-order path	0.1
LPOP	weight of odd-length path	$\frac{1}{\lambda_{max}}$
RPCA	weighting parameter	0.3

## Data Availability

We have provided complete references and URLs for all datasets used in the manuscript, and the data are publicly accessible via these cited sources.
